# The MCM3AP-AS1/miR-126/VEGF axis regulates cancer cell invasion and migration in endometrioid carcinoma

**DOI:** 10.1186/s12957-021-02316-0

**Published:** 2021-07-13

**Authors:** Jie Yu, Qiqi Fan, Lingling Li

**Affiliations:** 1 Department of Gynecology, The Affiliated Hospital of Qingdao University, Qingdao City, Shandong Province 266003 People’s Republic of China; 2 Department of Liver Diseases, The Sixth People’s Hospital of Qingdao, Qingdao City, Shandong Province 266000 People’s Republic of China; 3 Department of Reproductive Medicine, Qingdao Municipal Hospital, No.5 Donghai Road Shinan District, Qingdao City, Shandong Province 266071 People’s Republic of China

**Keywords:** Endometrioid carcinoma, MCM3AP-AS1, Survival, miR-126, VEGF

## Abstract

**Background:**

Long non-coding RNA (lncRNA) MCM3AP-AS1 plays an oncogenic role in several malignancies, but its role in endometrioid carcinoma (EC) is unclear. This study was carried out to explore the role of MCM3AP-AS1 in EC.

**Methods:**

A total of 60 EC patients were enrolled in this study. Expression levels of MCM3AP Antisense RNA 1 (MCM3AP-AS1), microRNA-126 (miR-126), and vascular endothelial growth factor (VEGF) in tissues and transfetced cells were measured by RT-qPCR. Cell transfections were performed to explore the interaction among MCM3AP-AS1, miR-126 and VEGF. Transwell assays were perfromed to evaluate the invasion and migration abilities of HEC-1 cells after transfection.

**Results:**

MCM3AP-AS1 was upregulated in EC and predicted poor survival. MCM3AP-AS1 directly interacted with miR-126. In EC cells, overexpression of MCM3AP-AS1 and miR-126 did not significantly affect the expression of each other. In addition, overexpression of MCM3AP-AS1 increased the expression levels of VEGF, a target of miR-126. Moreover, overexpression of MCM3AP-AS1 and VEGF increased the migration and invasion rates of EC cells, while overexpression of miR-126 suppressed these cell behaviors. Overexpression of MCM3AP-AS1 attenuated the role of miR-126 in cell invasion and migration.

**Conclusions:**

Therefore, MCM3AP-AS1 may serve as a competing endogenous RNA (ceRNA) of miR-126 to upregulate VEGF, thereby regulating cancer cell behaviors in EC.

## Background

As a type of cancer originates from gynecologic system, endometrioid carcinoma (EC) is one of the most commonly diagnosed malignancies in women [1]. It has been reported that more than 2.6% of women in the USA experience EC during their lifetime [2, 3]. In recent years, an increasing number of young females are also diagnosed with EC [2, 3]. As a consequence, female fertility is significantly affected in these patients [4]. The history of polycystic ovarian syndrome, nulliparity, and obesity are main risk factors for the occurrence and development of EC [5], while the molecular pathogenesis of this disease is still largely unknown. Although most EC patients (about 75%) are diagnosed at early stages, distant tumor metastasis is common [6]. Once metastasis has occurred, the prognosis will be extremely poor. Recently, a molecular tool based on the analysis of a multi-gene NGS panel has been developed to classify EC prognostic biotypes, while the accuracy and reliability remain to be verified [7]. Therefore, novel approaches are needed to prevent and treat cancer metastasis.

Studies have characterized various molecular players involved in almost all aspects of EC [1, 8]. Elucidation of the functions of these molecular factors has provided novel insights into the development of novel anti-EC therapies, such as targeted therapy [9, 10]. However, the functions of most molecular players remain to be identified. Long (> 200 nt) non-coding RNAs (lncRNAs) participate in cancer biology mainly by regulating gene expression rather than coding proteins [11]. Therefore, lncRNAs are potential targets for cancer treatment [11]. LncRNA MCM3AP Antisense RNA 1 (MCM3AP-AS1) plays an oncogenic role in several types of cancer, such as hepatocellular carcinoma, papillary thyroid cancer, glioblastoma, and cervical cancer [12–15]. It has been well established that MCM3AP-AS1 participates in cancer biology mainly by sponging tumor suppressive miRNAs to upregulate oncogene expression [12–15]. However, the role of MCM3AP-AS1 in EC is unknown. We predicted that MCM3AP-AS1 could interact with microRNA-126 (miR-126), which plays a tumor suppressive role mainly by downregulating vascular endothelial growth factor (VEGF) [16]. Moreover, our preliminary sequencing analysis also revealed the altered expression of MCM3AP-AS1 in EC (data not shown). Therefore, we may speculate that MCM3AP-AS1 may participate in EC and interact with miR-126. The interactions among MCM3AP-AS1, miR-126, and VEGF were also explored in EC.

## Methods

### EC patients and specimens

This study was approved by the Ethics Committee of Qingdao Municipal Hospital (No. 1265QMM#3233). A total of 60 EC patients (39 to 66 years old, mean age 51.9 ± 6.3 years old) were enrolled at this hospital between December 2012 and December 2014. All patients were newly diagnosed EC cases, and no therapy was initiated before this study. During biopsy (fine needle aspiration), EC tissues and paired non-tumor tissues (normal endometrial tissues within 5 cm around tumors) from each patient were collected. Tissues were confirmed by histopathological exam. All participates signed the written informed consent.

### A 5-year follow-up

According to the American Joint Committee on Cancer staging system, the 60 EC patients included 11, 18, 20, and 11 cases at stages I–IV, respectively. Therapies were determined based on AJCC stages. Patients were visited every month for a total of 5 years from the day of admission. Follow-up was performed through outpatient visit. Cancer conditions were assessed through CT scans, CA 125 blood test, and/or biopsies. The survival of patients was recorded. Patients died of causes other than EC were excluded from this study.

### Cell transfections

Human EC cell line HEC-1 (ATCC, Manassas, VA, USA) was used in this study. HEC-1 cells were cultivated in DMEM (10% FBS) in an incubator at 37 °C with 5% CO_2_ and 95% humidity. Expression vector of MCM3AP-AS1 (NCBI Accession: NR_110565.1) or VEGF (NCBI Accession: M32977.1) was established by inserting cDNAs into pcDNA3.1 vector (Invitrogen, Shanghai, China). Mimic of miR-126 (5′-UCGUACCGUGAGUAAUAAUGCG-3′) and the miRNA negative control (NC, 5′-UGUCUAGUCGUACCCAGUAGCG-3′) were purchased from Invitrogen. Transfections were performed using lipofectamine 2000 reagent (Invitrogen, Shanghai, China) to transfect 10 nM expression vector or 45 nM miRNA into 10^7^ HEC-1 cells. Un-transfected HEC-1 cells were used as the control (C) cells. Empty vector or miRNA NC-transfected HEC-1 cells were used as NC cells. Cells were cultivated in fresh medium for another 48 h prior to the subsequent assays.

### RNA interaction prediction

The direct interaction between MCM3AP-AS1 and miR-126 was predicted by IntaRNA 2.0 (http://rna.informatik.uni-freiburg.de/IntaRNA/Input.jsp). MiR-126 was inputted as the short sequence and MCM3AP-AS1 was inputted as the long sequence. All other parameters were default.

### Dual luciferase reporter assay

Luciferase vector of MCM3AP-AS1 (full length) was constructed using pGL3 vector (Promega Corporation, Madison, WI, USA) as backbone. In this vector, MCM3AP-AS1 was fused with the DNA coding sequence for luciferase gene. Transfections were performed with aforementioned methods to co-transfect MCM3AP-AS1 + miRNA NC (NC group) or MCM3AP-AS1 vector + miR-126 mimic (miR-126 group) into HEC-1 cells. Luciferase activity was detected after 48 h.

### RNA samples and RT-qPCR assays

Total RNAs were isolated from HEC-1 cells (10^8^) and paired tissues (0.02 g) using RNAzol. miRNAs were precipitated using 85% ethanol. Genomic DNAs were removed from RNA samples by digestion with gDNA eraser (Takara) until an OD 260/280 ratio close to 2.0 (pure RNA) was reached. RNA samples were reverse transcribed into cDNAs using SSRT IV system (Thermo Fisher Scientific, Waltham, MA, USA). With cDNAs as template, PowerUp SYBR™ Green Master Mix (Thermo Fisher Scientific, Waltham, MA, USA) was used to prepare all qPCR reactions. The expression levels of MCM3AP-AS1 and VEGF were determined with GAPDH as the endogenous control. The expression levels of mature miR-126 were measured using All-in-One™ miRNA qRT-PCR Detection Kit (Genecopoeia, Guangzhou, China) following the manufacturer’s instructions. The internal control for miR-126 was U6. Three replicates were performed and the gene expression levels were normalized using the 2^−ΔΔCT^ method. PCR reaction conditions were as follows: 95 °C for 1 min, and then 40 cycles of 95 °C for 12 s and 58 °C for 55 s. Primer sequences were as follows: 5′-GCTGCTAATGGCAACACTGA-3′ (forward) and 5′-AGGTGCTGTCTGGTGGAGAT-3′ (reverse) for MCM3AP-AS1; 5′-CTCCGAAACCATGAACTTT-3′ (forward) and 5′-CCACTTCGTGATGATTCTG-3′ (reverse) for VEGF; 5′-CAGGAGGCATTGCTGATGAT-3′ (forward) and 5′-GAAGGCTGGGGCTCATTT-3′ (reverse) for GAPDH. Forward primer of miR-126 was 5′-UCGUACCGUGAGUAAUAAUG-3′. The universal reverse primer and U6 forward primer were from the kit.

### Western blot

Total proteins were isolated from HEC-1 cells using RIPA solution. Protein concentrations were determined using BCA assay (Invitrogen, Shanghai, China). Protein samples were denatured in boiling water for 12 min, followed by protein separation using SDS-PAGE gel (8%). Proteins were transferred onto PVDF membranes, which were then blocked in PBS with 5% non-fat milk. Next, the membranes were incubated with rabbit primary antibodies of GAPDH (ab9845, 1:1,000, Abcam, Cambridge, UK) and VEGF (ab46154, 1:1,000, Abcam), followed by incubation with the HRP secondary antibody (ab6721, 1:1,000, Abcam). ECL (Invitrogen, Shanghai, China) was then used to develop signals.

### Cell migration and invasion assays

Transwell inserts (8 μm pore, Corning Inc. Corning, NY, USA) were used to evaluate the invasion and migration abilities of HEC-1 cells after transfection. Briefly, 6000 cells in 0.1 ml medium (serum-free) were transferred to the upper chamber, and the lower chamber was filled with medium containing 20% FCS. Matrigel (356234, Millipore, Burlington, MA, USA)-coated and uncoated membranes were used for invasion and migration assay, respectively. Cells were cultivated at 37 °C for 12 h. The upper surface of membrane was cleaned and the lower surface was stained with 1% crystal violet (Sigma-Aldrich, St. Louis, MO, USA). Cells were observed and counted under a light microscope.

### Statistical analyses

Unpaired t test and paired t test were used to compare gene expression and relative luciferase units for two independent groups and paired tissues, respectively. Comparisons among multiple groups were performed by ANOVA Tukey’s test. The 60 EC patients were grouped into two groups (high and low, n = 30, cutoff value = median expression level of MCM3AP-AS1 in EC). The 5-year follow-up data were used to plot survival curves. Chi-squared test was used to analyze the correlations between MCM3AP-AS1 and patients’ clinical data. *P* < 0.05 was considered as statistically significant.

## Results

### MCM3AP-AS1 was upregulated in EC and was correlated with poor survival

**Fig. 1:**
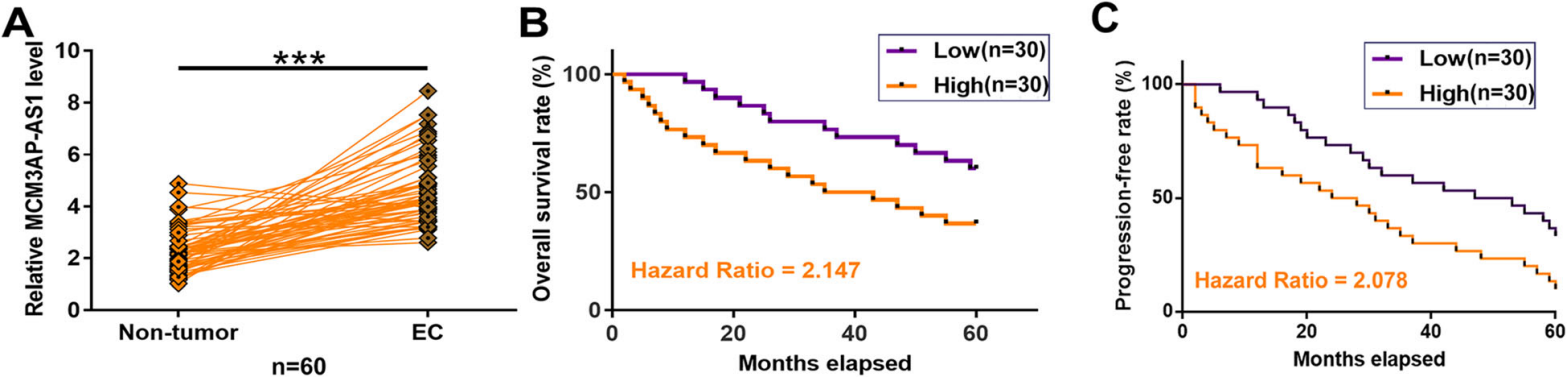
MCM3AP-AS1 was upregulated in EC and predicted poor survival. Paired EC and non-tumor tissues from EC patients (n = 60) were subjected to RNA preparations and RT-qPCR to analyze the expression of MCM3AP-AS1 (**A**). ***p < 0.001. The associations of MCM3AP-AS1 with EC patients’ overall survival (**B**) and progression-free survival (**C**) were analyzed by a 5-year follow-up study

RT-qPCR analysis showed that the expression levels of MCM3AP-AS1 were significantly higher in EC tissues compared to that in non-tumor tissues (Fig. 1A, *p* < 0.001). Survival curves were plotted and compared between the high and low MCM3AP-AS1 level groups. It was observed that high expression levels of MCM3AP-AS1 were closely correlated with poor overall survival (Fig. 1B) and progression-free rate (Fig.1C). Chi-squared test analysis revealed that expression levels of MCM3AP-AS1 were not significantly correlated with patients’ age, AJCC stage, tumor grade, BMI, smoking habit, pathology classification, grade, endocervical invasion, ovarian involvement, parametrium involvement, and lymph nodes metastasis (Table 1).

**Table 1:** Correlations between expression levels of MCM3AP-AS1 and patients’ clinical data

Items	Groups	Cases	High-expression	Low-expression	χ^2^	p value
Age	≥ 50	32	14	18	1.07	0.3
	< 50	28	16	12		
AJCC	I	11	5	6	1.93	0.59
	II	18	10	8		
	III	20	8	12		
	IV	11	7	4		
Grade	1	17	9	8	0.1	0.95
	2	23	11	12		
	3	20	10	10		
BMI	≥ 24	26	12	14	0.27	0.6
	< 24	34	18	16		
Smoking	Yes	18	7	11	1.27	0.26
	No	42	23	19		
Pathology classification	Well+Mod	40	21	19	0.3	0.58
	Poor	20	9	11		
Grade	G1	24	14	10	1.11	0.29
	G2/3	36	16	20		
Endocervical invasion	Yes	44	23	21	0.34	0.56
	No	16	7	9		
Ovarian involvement	Yes	22	13	9	1.15	0.28
	No	38	17	21		
Parametrium involvement	Yes	18	11	7	1.27	0.26
	No	42	19	23		
Lymph nodes metastasis	Yes	15	7	8	0.09	0.77
	No	45	23	22		

### The interaction between MCM3AP-AS1 and miR-126 showed no effect on the expression of each other

**Fig. 2:**
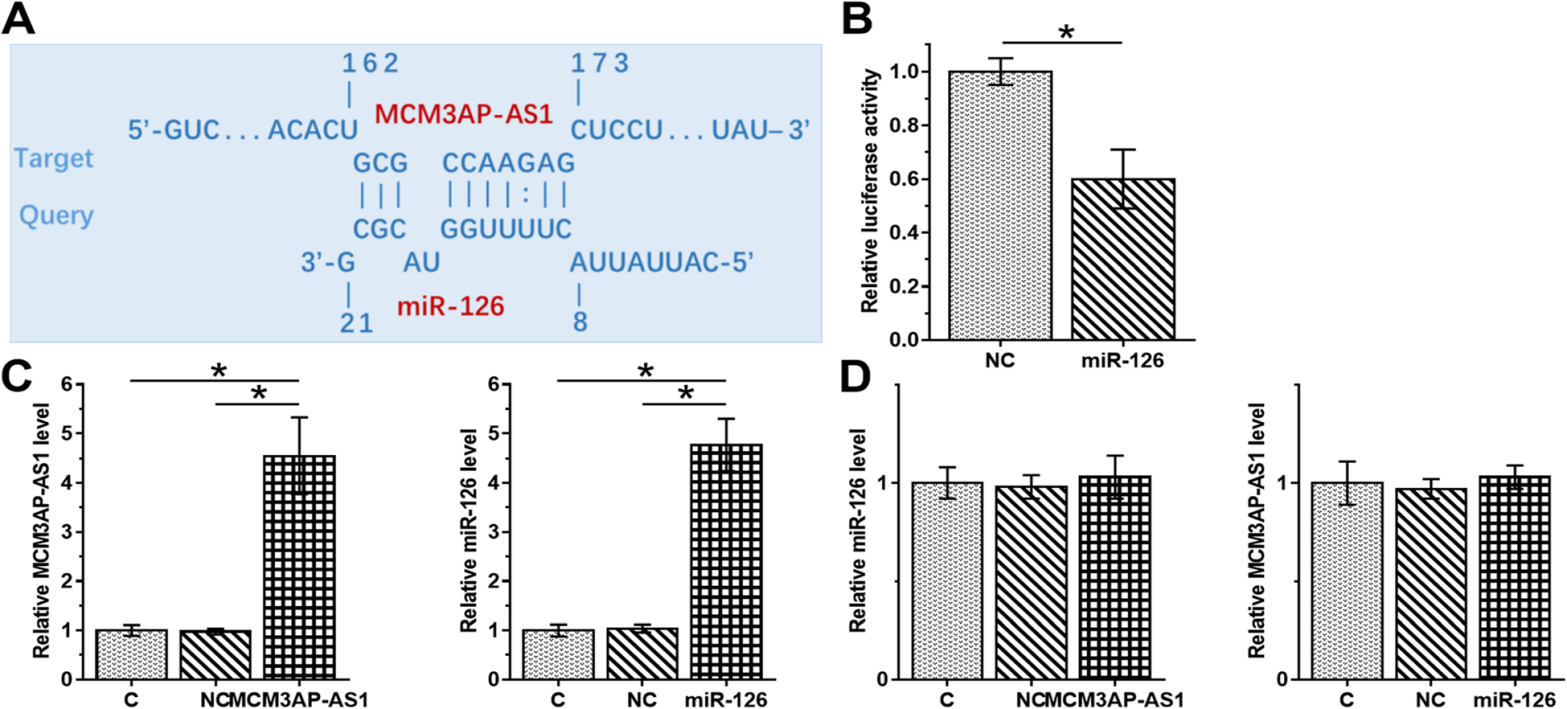
The interaction between MCM3AP-AS1 and miR-126 showed no significant effect on the expression of each other. The direct interaction between MCM3AP-AS1 and miR-126 was predicted by IntaRNA 2.0 (http://rna.informatik.uni-freiburg.de/IntaRNA/Input.jsp) (**A**). Dual luciferase assay was performed by co-transfecting cells with MCM3AP-AS1 + miRNA NC (NC group) or MCM3AP-AS1 vector + miR-126 mimic (miR-126 group). Luciferase activity was measured and compared at 48 h post-transfection (**B**). MCM3AP-AS1 and miR-126 were overexpressed (**C**) to study the effects on the expression of each other (**D**). *p < 0.05

IntaRNA2.0 [17] prediction revealed that MCM3AP-AS1 and miR-126 could form strong base pairing (Fig. 2A). Dual luciferase assay showed that, compared to NC group, luciferase activity was significantly lower in miR-126 group, indicating that MCM3AP-AS1 and miR-126 might interact with each other (Fig. 2B, *p* < 0.05). To further confirm the interaction between them, MCM3AP-AS1 and miR-126 were overexpressed (Fig. 2C, *p* < 0.05). Overexpression of MCM3AP-AS1 and miR-126 showed no effect on the expression of each other (Fig. 2D).

### Overexpression of MCM3AP-AS1 increased the expression levels of VEGF

**Fig. 3:**
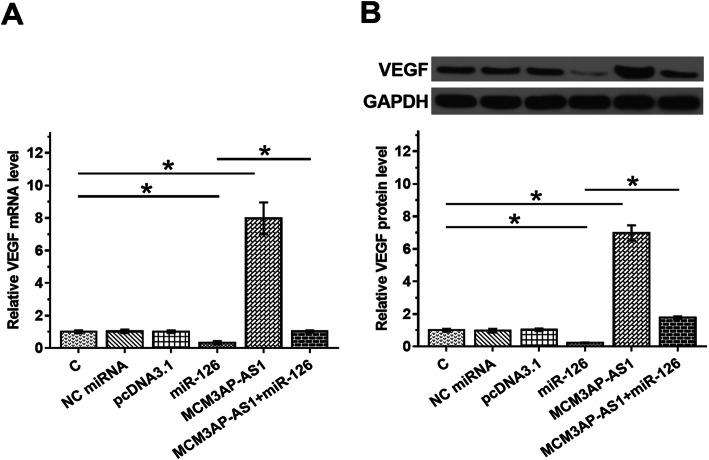
Overexpression of MCM3AP-AS1 increased VEGF expression. The expression of VEGF, a target of miR-126, in cells with MCM3AP-AS1 or miR-126 expression was analyzed by RT-qPCR (**A**) and western blot (**B**). *p < 0.05

The expression of VEGF, a target of miR-126, in cells with overexpression of MCM3AP-AS1 or miR-126 was evaluated by RT-qPCR (Fig. 3A) and western blot (Fig. 3B). Overexpression of miR-126 decreased the expression levels of VEGF mRNA (Fig. 3A, *p* < 0.05). Western blot data also showed that overexpression of miR-126 decreased the expression levels of VEGF protein (Fig. 3B, *p* < 0.05). These data confirmed the targeting of VEGF by miR-126. Overexpression of MCM3AP-AS1 increased the expression levels of VEGF and attenuated the effects of overexpression of miR-126 (*p* < 0.05).

### MCM3AP-AS1 promoted HEC-1 cell invasion and migration through the miR-126/VEGF axis

**Fig. 4:**
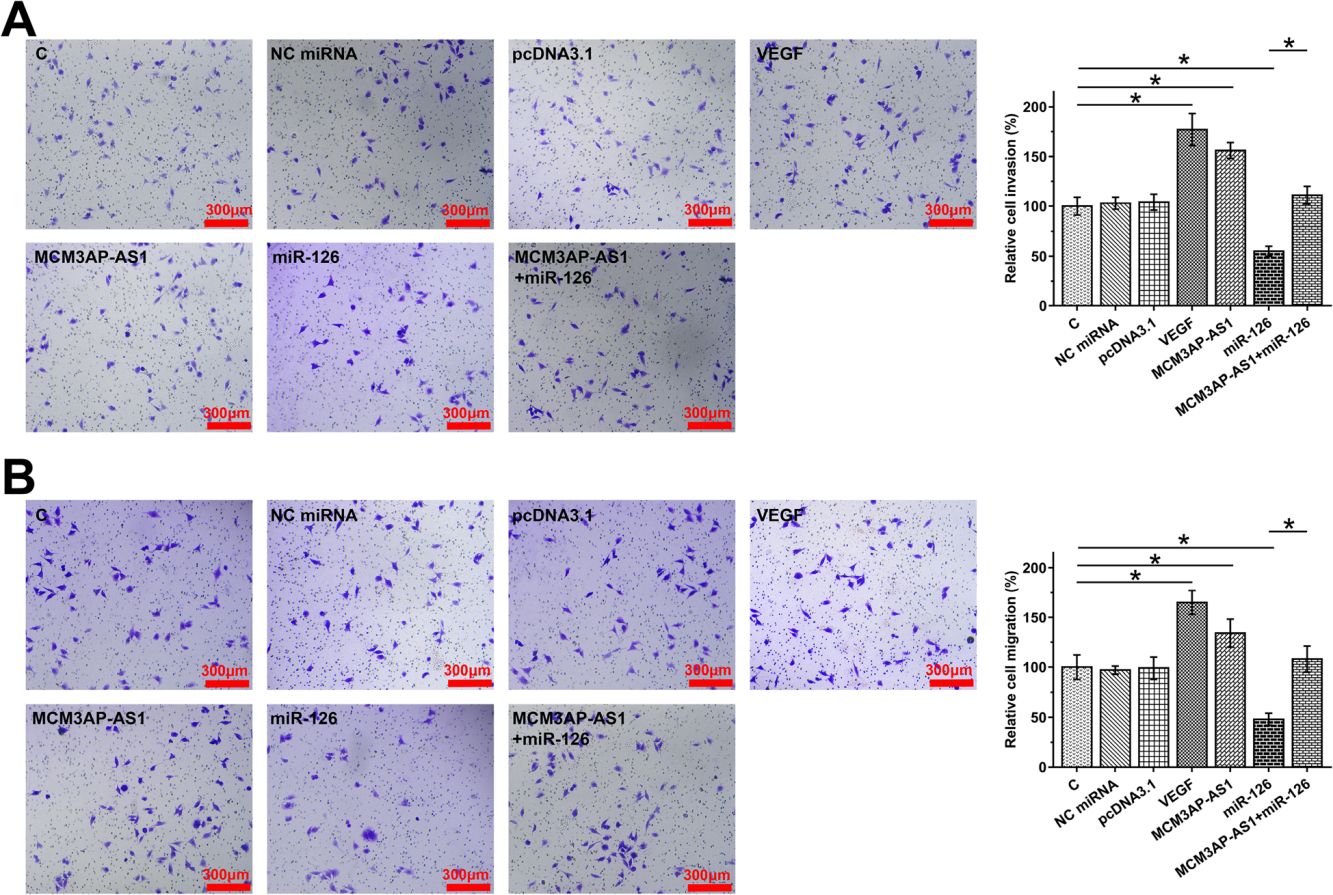
MCM3AP-AS1 promoted HEC-1 cell invasion and migration through the miR-126/VEGF axis. The effects of transfections on the invasion and migration of HEC-1 cells were analyzed by Transwell invasion (**A**) and migration (**B**) assay. *p < 0.05

Transwell assay results showed that overexpression of MCM3AP-AS1 and VEGF increased the migration (Fig. 4A) and invasion (Fig. 4B) rates of HEC-1 cells, while overexpression of miR-126 suppressed cell behaviors. In addition, overexpression of MCM3AP-AS1 suppressed the role of miR-126 (*p* < 0.05).

## Discussion

We reported that the MCM3AP-AS1/miR-126/VEGF axis was a novel pathway involved in the pathogenesis of EC. We found that MCM3AP-AS1 was upregulated in EC and might play its oncogenic role by regulating cell behaviors through the miR-126/VEGF axis.

The oncogenic role of MCM3AP-AS1 has been reported in several types of cancer [12–14]. During the development of hepatocellular carcinoma, MCM3AP-AS1 is upregulated and interacts with the miR-194-5p/FOXA1 axis to promote tumor growth [12]. MCM3AP-AS1 is also upregulated in glioblastoma and regulates angiogenesis through the miR-211/KLF5/AGGF1 axis [13]. MCM3AP-AS1 was downregulated in papillary thyroid cancer and could serve as a ceRNA of miR-211-5p to upregulate SPARC, thereby aggregating disease conditions [14]. In this study, we showed that MCM3AP-AS1 was also upregulated in EC and its overexpression resulted in increased migration and invasion rates of EC cells, suggesting that MCM3AP-AS1 may also play an oncogenic role in EC.

In addition, we found that the poor survival of EC patients was correlated with the high expression levels of MCM3AP-AS1. With the development of detection techniques, most EC patients (about 75%) are diagnosed at early stages, while metastatic disease is frequently diagnosed [6, 18]. Accurate prognosis may improve the survival of EC patients by guiding the determination of treatment approaches. Therefore, monitoring the expression levels of MCM3AP-AS1 may assist the prognosis of EC, thereby promoting the survival of patients. However, the reliability of using MCM3AP-AS1 as a prognostic marker for EC remains to be further assessed.

It has been reported that miR-126 can target insulin receptor substrate 1 to suppress the invasion and migration of cancer cells [19]. In this study, we showed that miR-126 may downregulate VEGF to inhibit EC cell invasion and migration. Moreover, we showed that MCM3AP-AS1 may serve as a ceRNA of miR-126 to upregulate VEGF. Previous studies reported that MCM3AP-AS1 could serve as a ceRNA of different miRNAs (such as miR-194-5p and miR-211) in different cancers [12–14]. Therefore, we speculated that MCM3AP-AS1 may play oncogenic roles in cancer biology mainly by serving as a ceRNA of tumor suppressive miRNAs. In addition, a recent study showed that MCM3AP-AS1 was downregulated in cervical cancer and it could downregulate miR-93 to inhibit cell proliferation in cervical cancer [15]. Therefore, MCM3AP-AS1 may play different roles in different cancers. It is worth noting that, in this study, no significant difference was observed in the expression levels of MCM3AP-AS1 in different histological types (mucinous carcinoma, serous carcinoma, clear cell carcinoma, squamous cell carcinoma, and undifferentiated carcinoma in this study) and molecular subtypes (POLE ultramutated, microsatellite instability hypermutated, copy number low, and copy number high in this study). This is possibly due to the small sample size. The differential expression of MCM3AP-AS1 in different histological types and molecular subtypes of EC remain to be explored.

Our study is the first to report the role of MCM3AP-AS1 in EC and characterize a novel MCM3AP-AS1/miR-126/VEGF pathway in the invasion and migration of EC cells. Therefore, MCM3AP-AS1 is likely a potential target to treat EC. However, this study is limited by the small number of patients and the lack of in vivo characterization of gene functions. Future studies are still needed to confirm the role of MCM3AP-AS1 in EC.

It is worth noting that previous studies have identified multiple lncRNAs and miRNAs with critical functions in EC and other cancers [20–26]. Future studies should focus on the clinical applications of these lncRNAs and miRNAs.

## Conclusions

In conclusion, MCM3AP-AS1 is upregulated in EC and may promote cancer cell invasion and migration by serving as a ceRNA of miR-126 to upregulate VEGF. In this study, we characterized a novel MCM3AP-AS1/miR-126/VEGF pathway in EC. This novel pathway may serve as a potential target to treat EC. However, clinical trials are needed to confirm our hypothesis.

## Data Availability

We accept reasonable request.

## Abbreviations

EC: Endometrial carcinoma
NC: Negative control
C: Control
lncRNAs: Long non-coding RNAs
VEGF: Vascular endothelial growth factor
